# Hereditary or acquired? Comprehensive genetic testing assists in stratifying angioedema patients

**DOI:** 10.1186/s13223-024-00889-5

**Published:** 2024-03-30

**Authors:** Marija Rozevska, Adine Kanepa, Signe Purina, Linda Gailite, Inga Nartisa, Henriette Farkas, Dmitrijs Rots, Natalja Kurjane

**Affiliations:** 1 Riga Stradiņš University, Riga, Latvia; 2 Children’s clinical university hospital, Riga, Latvia; 3 Pauls Stradiņš Clinical University Hospital, Riga, Latvia; 4 Center of Diagnostics and Treatment of Allergic Diseases, Riga, Latvia; 5 Department of Internal Medicine and Haematology, Hungarian Angioedema Center of Reference and Excellence, Semmelweis University, Budapest, Hungary

**Keywords:** Diagnostic challenges, Unresolved cases, Exome sequencing, Genome sequencing, Diagnostic yield

## Abstract

Hereditary angioedema (HAE) poses diagnostic challenges due to its episodic, non-specific symptoms and overlapping conditions. This study focuses on the genetic basis of HAE, particularly focusing on unresolved cases and those with normal C1-inhibitor levels (nC1-INH HAE). This study reveals that conventional testing identified pathogenic variants in only 10 patients (*n* = 32), emphasizing the necessity for an integrative approach using genome, exome, and transcriptome sequencing. Despite extensive genetic analyses, the diagnostic yield for nC1-INH HAE remains low in our study, the pathogenic variant for nC1-INH HAE was identified in only 1 patient (*n* = 21). Investigation into candidate genes yielded no pathogenic variants, prompting a re-evaluation of patients’ diagnoses. This study advocates for a nuanced approach to genetic testing, recognizing its limitations and emphasizing the need for continuous clinical assessment. The complex genetic landscape of nC1-INH HAE necessitates further research for a more comprehensive understanding. In conclusion, this study contributes valuable insights into the genetic intricacies of HAE, highlighting the challenges in diagnosis and the evolving nature of the disease. The findings underscore the importance of advanced sequencing techniques and an integrated diagnostic strategy in unravelling the complexities of HAE, particularly in nС1-INH HAE cases.

**Supplementary Information:**

The online version contains supplementary material available at 10.1186/s13223-024-00889-5.

## Introduction

Hereditary angioedema (HAE) is a rare [1] monogenic disorder characterised by spontaneous or provoked angioedema attacks affecting different body parts such as the face, upper airways, limbs, genitals, and gastrointestinal tract. HAE can be life-threatening, especially if oedema affects the upper airways leading to asphyxiation [2, 3] In most of bradykinin causes blood vessel leakage, leading to localised swelling and angioedema in various body parts [4].

HAE is classified into three main types: HAE-I, HAE-II, and HAE with normal C1-inhibitor (nC1-INH HAE). Each type is associated with specific genetic variations and clinical manifestations. HAE-I is the most common form, accounting for approximately 80–85% of cases [3, 5]. It is characterised by reduced levels of functional and antigenic levels of a C1 inhibitor (C1-INH) in the blood due to pathogenic variants in the *SERPING1* gene. HAE-II represents approximately 10–15% [1] of C1-INH cases, is characterised by dysfunctional C1-INH production with normal blood levels, and is caused by protein-altering variants (e.g. missense) in the C1-INH protein’s active centre. However, despite known monogenic aetiology affecting a single gene, approximately 5–15% of the HAE-I and II cases remain molecularly unsolved [6–9]. This is partially explained by limitations of current technologies, e.g. missing deep-intronic variants [10, 11] structural variants, and partially in our limited ability to interpret novel variants.

nC1-INH HAE is the rarest form of HAE and accounts for < 5% of cases [12]. In recent years, extensive research has been conducted to further elucidate the underlying genetic mechanisms and pathophysiology of nC1-INH HAE [13], though most cases remain unknown [14]. Multiple genes have been proposed such as coagulation factor XII (*F12*), plasminogen (*PLG*), angiopoietin 1 (*ANGPT1*), kininogen 1 (*KNG1*), myoferlin (*MYOF*), and heparan sulfate-glucosamine 3-sulfotransferase 6 (*HS3ST6*); however, except for *F12* and *PLG*, the reports are usually limited to a single family [13, 15] without confirmation in unrelated families, thus limiting such candidate gene testing in diagnostic settings [16].

Diagnosing HAE can be challenging due to several factors. First, symptoms are usually non-specific and overlap with other acquired conditions. Phenocopies, which are conditions that mimic HAE symptoms, are common, leading to misdiagnosis and delayed treatment. These phenocopies include acquired angioedema, allergic angioedema, and idiopathic angioedema [15]. Distinguishing HAE from phenocopies will help to find appropriate treatment for patients, thus avoiding unnecessary treatment. Establishing the correct diagnosis of HAE helps to prevent complications and improve quality of life for the patients [17], therefore, it is essential to differentiate HAE from other conditions by applying a thorough clinical evaluation and laboratory investigations. Second, HAE symptoms are episodic and may not manifest until later in life, making it challenging to identify the condition in childhood. Finally, due to its rarity and complexity, many healthcare professionals may have a limited awareness of HAE or its diagnostics, which can lead to misdiagnosis or delays in appropriate management [18].

Our study aimed to elucidate the genetic basis of HAE, focusing on unsolved cases and cases with normal C1-INH levels. Utilizing genome, exome, and transcriptome sequencing, we identified previously unidentified pathogenic variants, highlighted the limitations of traditional sequencing methods, clarified the classification of a previously-identified variant, and emphasised the need for ongoing exploration and clinical re-evaluation in nC1-INH HAE cases.

## Results and discussion

In our study, a cohort of 32 patients were enrolled, comprising nine patients presenting with clinical indications of HAE-I (both C1-INH and C1-INH activity were significantly low), one healthy relative of a proven HAE-I patient, one patient suspected of HAE-II (C1-INH activity was low with typical clinical signs), and 21 patients with suspected nC1-INH HAE. Employing conventional genetic testing methodologies (Sanger sequencing of *SERPING1*, *PLG*, and *F12* genes) as described before [19], (likely) pathogenic genetic variants were successfully identified in eight (in seven out of nine investigated patients with diagnosed HAE-I and one out of 21 patients with nC1-INH-HAE) out of the 32 patients. Additionally, one patient with HAE-I had an intronic/splice region variant of uncertain significance in the *SERPING1* gene. Patient 32, herein referred to as the relative of Patient 15, initially underwent testing as a healthy family member; however, the analysis revealed the presence of a pathogenic variant in the *SERPING1* gene. Subsequent assessments demonstrated diminished levels of C1N and reduced C1 activity, leading to the asymptomatic diagnosis of HAE-I.

### Genome sequencing provides definitive diagnosis and high confidence to re-evaluate clinical diagnosis

Interestingly, the initial genetic testing failed to identify a pathogenic *SERPING1* variant in three patients with suspected HAE-I or HAE-II (patient no. 8 had low levels of C1-INH ag, very low C1-INH activity, and low levels of C4; patient no. 22 also had low levels of C1-INH ag, low C1-INH activity, and low levels of C4; patient no. 11 had normal levels of C1-INH ag, but mildly decreased levels of C1-INH activity, and low C4). All tests were performed twice. Therefore, we performed genome sequencing for these three patients focusing first on coding SNVs (single nucleotide variants) and CNVs (copy number variations) in *SERPING1*, following by analysis of other SVs (structural variants) and non-coding SNVs. We were able to detect a pathogenic deletion of exon 4 with exact breakpoints at chr11[GRCh38]:g.57600729_57603011del (Figure S1) in one patient (Patient no. 22), confirming the diagnosis of HAE-I for this individual. The variant was not identified previously because MLPA (Multiplex ligation-dependent probe amplification) was not routinely applied for such cases.

In contrast, no pathogenic variants were found in the other two patients (patient no. 8 and 11) even after analysis of SVs and non-coding gene parts, prompting a re-evaluation of their diagnoses. After a two-year follow-up, in one of these patients (Patient no. 8) that had no family history of angioedema, paraproteins (monoclonal IgM) were eventually detected in the blood test of this patient, which were not present at the initial assessment two years prior. This led to confirming an alternative aetiology for the symptoms. Remarkably, the symptoms of another patient (Patient no. 11) with suspected HAE-II and no family history of angioedema resolved at follow-up, and the function of C1 activity returned to normal (C1-INH activity was mildly decreased twice), necessitating a revision of the diagnosis. These laboratory abnormalities could be related to the fact that the samples were shipped to another country for analysis, thus, longer shipping times. The patient is now considered to have bradykinin-related angioedema (antihistamine medications were found ineffective and other potential causes of angioedema were ruled out). Therefore, we show that genome sequencing can not only solve previously-unsolved cases, but also provide high confidence of an alternative cause for the “negative” cases.

### Transcriptome sequencing provides intronic variant re-classification

**Fig. 1:**
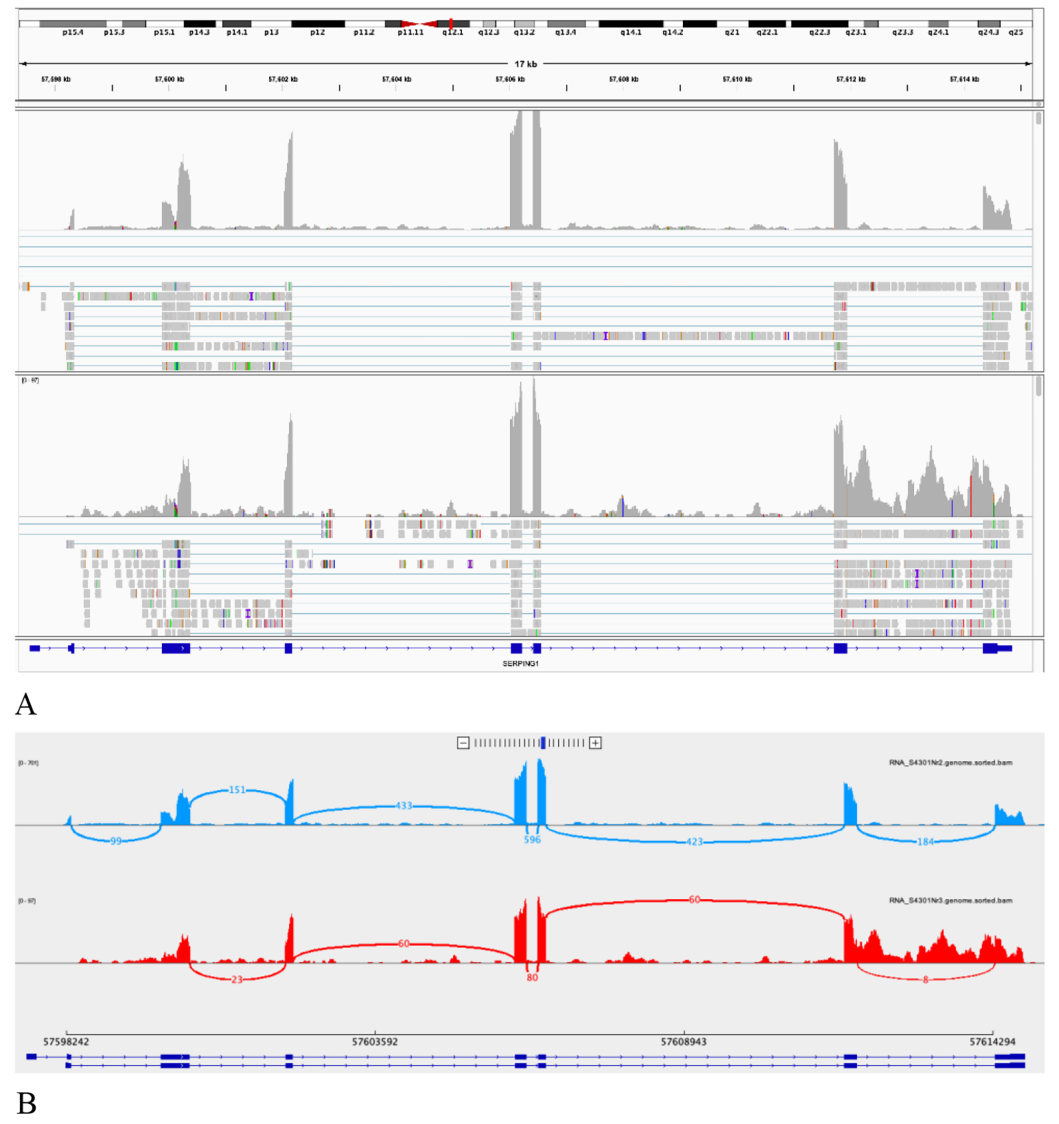
Retention of the last *SERPING1* intron present due to the NM_000062.2:c.1249 + 4 A > G r.spl variant found in patient no. 9 using transcriptome sequencing. (A) cDNA sequence alignment to the genome in control (top) and patient (bottom) samples. Note increased coverage of a single allele of the last intron. (B) Sashimi plot of the splicing site usage in control (top) and patient (bottom) samples. Note low number reads covering the canonical splice site between the last two exons

Furthermore, among the patients diagnosed with HAE-I, one individual (Patient no. 9) was found to have a novel intronic splice region variant in the *SERPING1* gene through Sanger sequencing NM_000062.2:c.1249 + 4 A > G, p?. The variant was predicted to result in a loss of canonical donor site (-4 bp) by Pangolin (splice loss = 0.71), but had low prediction confidence by spliceAI (donor loss = 0.16). Given the contradictory predictions and variant’s location in a non-canonical splice region, the actual effects were unknown. The segregation was limited to only one affected family member, therefore, without confirmation of a splicing defect, it was classified as a variant of uncertain significance. To evaluate the possible effects on splicing and assess the pathogenicity of this variant, whole transcriptome analysis with rRNA and globin depletion on peripheral blood-extracted RNA was performed. It revealed full intron retention on one allele in mRNA (with escaping nonsense mediated decay) (Fig. 1), successfully confirming the presence of a splicing defect due to the variant, allowing for the reclassification of the variant as pathogenic. This defect could easily be missed by targeted RT-PCR because the intron is ∼ 2.5Kb large and could fail the amplification.

### Genetic testing of all currently known HAE (candidate) genes has low yield for nC1-INH-HAE, but is necessary for precise clinical diagnosis

To further investigate the genetic basis of nC1-INH-HAE in the remaining 20 patients, we performed exome sequencing, focusing on the analysis of coding SNVs and CNVs in recently-described nC1-INH-HAE (candidate) genes *(F12, PLG, ANGPT1, KNG1, MYOF*, and *HS3ST6)*, as well other genes potentially involved in angioedema pathogenesis published previously [20] (total *n* = 55 genes; Table S1). However, no pathogenic or rare uncertain significance variants were detected in the genes of interest. Following these genetic analyses, all patients underwent a comprehensive re-evaluation of their clinical presentation, leading to a revision of their diagnoses.

Notably, none of the patients had a family history of angioedema attacks. As a result of the clinical re-evaluation after the negative result, 16 patients were ultimately diagnosed with bradykinin-related angioedema, while four patients were identified as having histaminergic angioedema since they reacted to treatment by Omalizumab.

In our study involving 32 patients with suspected HAE, encompassing various types and suspected nC1-INH HAE, conventional genetic testing identified pathogenic alterations in 10 patients. Genome sequencing for three unsolved patients revealed a previously-missed pathogenic variant, illustrating genome sequencing’s capacity to resolve challenging cases. Similarly Ren et al. [21]was able to solve all cases with HAE-I or HAE-II using genome sequencing for the initially-“negative” cases. Notably, the absence of pathogenic variants in two patients, despite extensive analysis, prompted a re-evaluation of their diagnoses; the emergence of paraproteins in one patient led to a revised diagnosis of histaminergic AE, while another patient’s symptoms resolved and their C1 activity normalised. Similarly, we were not able to identify a clinically-significant variant in 20 out of 21 nC1-INH HAE-suspected cases, even after exome sequencing with analysis of currently-confirmed and candidate HAE genes, which also resulted in a re-evaluation of their diagnosis (to different types of AE). This highlights the complex nature, symptom evolution, and variable clinical presentation of HAE and other angioedemas, and shows how even “negative” genetic results after comprehensive genetic testing can aid in reinterpretation of a clinical diagnosis. This dynamic nature of the disease challenges the static nature of genetic testing and highlights the importance of an integrative and continuous assessment of patient symptoms and genetic information.

Additionally, we highlight challenges in the interpretation of genetic results, particularly in the context of variants of uncertain significance. Functional validation of the variants to ascertain their clinical relevance is crucial, as exemplified by our reclassification of a novel *SERPING1* splicing variant. Our findings show how application of transcriptome sequencing can help the reclassification of variants of uncertain significance that affect splicing.

Our findings indicate that the current diagnostic yield of nC1-INH HAE remains low. While newly proposed candidate genes have been reported as potentially involved in the pathogenesis of nC1-INH-HAE, there is no evident clinical justification for conducting genetic testing of these genes. Except for a single case with a pathogenic variant in the *PLG* gene, no pathogenic variants were identified among 20 patients in any of the described genes. Moreover, even if such variants were to be detected, they would likely be classified as variants of uncertain significance due to the limited described genotypic spectrum, providing limited assistance in the clinical diagnosis of the patients. Recently, another study sequenced 55 gene panels for 133 suspected-HAE individuals and failed to identify additional molecular diagnoses [20]. Further research is necessary to validate the association of these genes with nC1-INH HAE. Therefore, based on our and previous study results, sequencing of the described (candidate) genes has limited utility and should not be currently applied in diagnostic settings, except for *F12* and *PLG*, which have a proven association with nC1-INH HAE. This experience underscores the complexity of the genetic landscape and the need for a more comprehensive understanding of the genetic factors contributing to nC1-INH HAE.

## Concluding remarks

In conclusion, our study contributes to the growing knowledge of HAE, particularly in nC1-INH-HAE. While genetic testing remains a valuable tool and can aid genetic, as well as acquired angioedema diagnosis, its application should be nuanced, recognizing its limitations and the need for integrated diagnostic strategies.

### Electronic supplementary material

Below is the link to the electronic supplementary material.

Supplementary Material 1

## Data Availability

The identified variants are provided in this manuscript. Raw sequencing data are not publicly available due to institutional ethical restrictions.

## Abbreviations

HAE: Hereditary angioedema
MLPA: Multiplex ligation dependent probe amplification
