# Supplementary material for: Hereditary or acquired? Comprehensive genetic testing assists in stratifying angioedema patients
Source: Allergy Asthma Clin Immunol. 2024 Mar 30;20:28. doi: 10.1186/s13223-024-00889-5 (PMC10981324; doi:10.1186/s13223-024-00889-5)
Supplement: Supplementary file 1 — Supplementary Material 1 [file 13223_2024_889_MOESM1_ESM.docx]

**Supplementary materials**


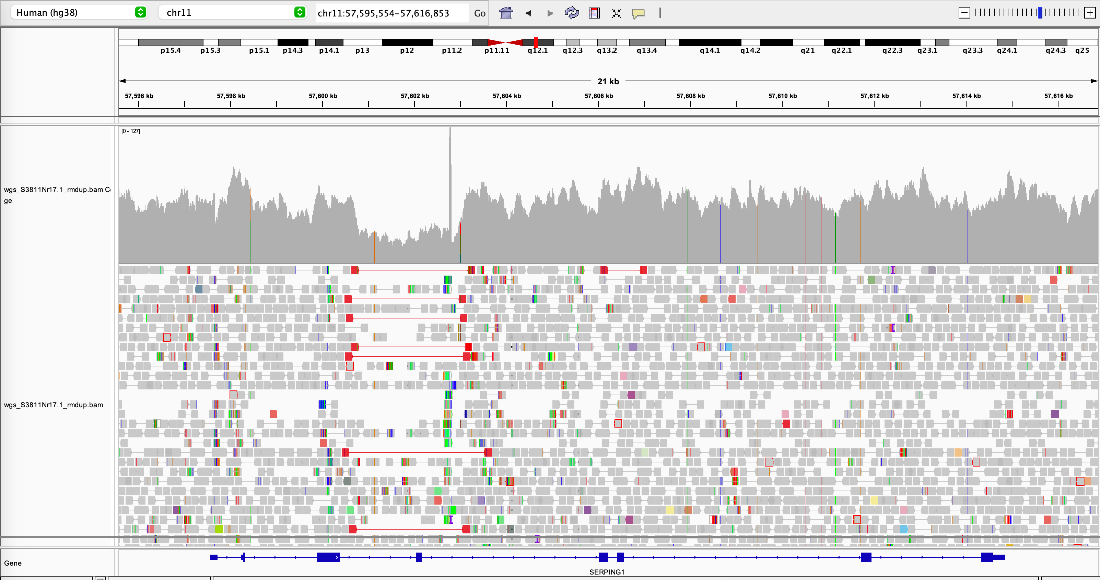


Figure S1. Exon 4 deletion identified in patient no. 22 using GS chr11[GRCh38]:g57600729_57603011del. Note the drop in the coverage and split reads over the 4th exon.

**Table S1**. Investigated patients with their initial clinical diagnosis of HAE and re-evaluated diagnosis after genetic testing results.

| **Patient no.** | **Initial diagnosis** | **Genetic finding** | **Current diagnosis** |
| --- | --- | --- | --- |
| 1 | nC1-INH HAE | Negative | Bradykinin-related AE |
| 2 | nC1-INH HAE | Negative | Bradykinin-related AE |
| 3 | nC1-INH HAE | Negative | Bradykinin-related AE |
| 4 | nC1-INH HAE | Negative | Bradykinin-related AE |
| 5 | HAE-I | NM_000062.2(*SERPING1*):c.1312del p.(Val438PhefsTer12) | HAE-I |
| 6 | HAE-I | NM_000062.2(*SERPING1*):c.1312del p.(Val438PhefsTer12) | HAE-I |
| 7 | nC1-INH HAE | Negative | Bradykinin-related AE |
| 8 | HAE-I | Negative | Acquired AE |
| 9 | HAE-I | NM_000062.2(*SERPING1*):c.1249+4A>G r.spl | HAE-I |
| 10 | HAE-I | NM_000062.2(*SERPING1*):c.1249+4A>G r.spl | HAE-I |
| 11 | HAE-II | Negative | Bradykinin-related AE |
| 12 | HAE-I | NM_000062.2(*SERPING1*):c.1396C>T p.(Arg466Cys) | HAE-I |
| 13 | nC1-INH HAE | Negative | Bradykinin-related AE |
| 14 | nC1-INH HAE | Negative | Bradykinin-related AE |
| 15 | HAE-I | NM_000062.2(*SERPING1*):c.1195C>T p.(Pro399Ser) | HAE-I |
| 16 | nC1-INH HAE | Negative | Bradykinin-related AE |
| 17 | nC1-INH HAE | Negative | Bradykinin-related AE |
| 18 | nC1-INH HAE | Negative | Bradykinin-related AE |
| 19 | nC1-INH HAE | Negative | Histaminergic AE |
| 20 | nC1-INH HAE | Negative | Bradykinin-related AE |
| 21 | nC1-INH HAE | Negative | Bradykinin-related AE |
| 22 | HAE-I | [GRCh38] chr11:g.57600729_57603011del | HAE-I |
| 23 | nC1-INH HAE | Negative | Histaminergic AE |
| 24 | nC1-INH HAE | Negative | Bradykinin-related AE |
| 25 | nC1-INH HAE | Negative | Histaminergic AE |
| 26 | nC1-INH HAE | Negative | Bradykinin-related AE |
| 27 | nC1-INH HAE | Negative | Bradykinin-related AE |
| 28 | nC1-INH HAE | Negative | Histaminergic AE |
| 29 | nC1-INH HAE | Negative | Bradykinin-related AE |
| 30 | HAE-I | NM_000062.2(*SERPING1*):c.550G>A p.(Gly184Arg) | HAE-I |
| 31 | nC1-INH HAE | NM_000301.5(*PLG*):c.988A>G p.(Lys330Glu) | PLG-nC1-INH-HAE |
| 32 | Healthy relative of HAE-I (N15) | NM_000062.2(*SERPING1*):c.1195C>T p.(Pro399Ser) | HAE-I |
